# Spot the difference: comparing results of analyses from real patient data and synthetic derivatives

**DOI:** 10.1093/jamiaopen/ooaa060

**Published:** 2020-12-14

**Authors:** Randi E Foraker, Sean C Yu, Aditi Gupta, Andrew P Michelson, Jose A Pineda Soto, Ryan Colvin, Francis Loh, Marin H Kollef, Thomas Maddox, Bradley Evanoff, Hovav Dror, Noa Zamstein, Albert M Lai, Philip R O Payne

**Affiliations:** 1 Division of General Medical Sciences, Department of Medicine, School of Medicine, Washington University in St. Louis, St. Louis, Missouri, USA; 2 Department of Medicine, Institute for Informatics, School of Medicine, Washington University in St. Louis, St. Louis, Missouri, USA; 3 Division of Pulmonary and Critical Care Medicine, Department of Medicine, School of Medicine, Washington University in St. Louis, St. Louis, Missouri, USA; 4 Division of Critical Care Medicine, Department of Anesthesiology and Critical Care Medicine, Children’s Hospital of Los Angeles, Los Angeles, California, USA; 5 School of Medicine, Washington University in St. Louis, St. Louis, Missouri, USA; 6 Healthcare Innovation Lab, BJC Healthcare, School of Medicine, Washington University in St. Louis, St. Louis, Missouri, USA; 7 MDClone Ltd, Beer Sheva, Israel

**Keywords:** synthetic data, protected health information, precision health care, electronic health records and systems, data analysis

## Abstract

**Background:**

Synthetic data may provide a solution to researchers who wish to generate and share data in support of precision healthcare. Recent advances in data synthesis enable the creation and analysis of synthetic derivatives as if they were the original data; this process has significant advantages over data deidentification.

**Objectives:**

To assess a big-data platform with data-synthesizing capabilities (MDClone Ltd., Beer Sheva, Israel) for its ability to produce data that can be used for research purposes while obviating privacy and confidentiality concerns.

**Methods:**

We explored three use cases and tested the robustness of synthetic data by comparing the results of analyses using synthetic derivatives to analyses using the original data using traditional statistics, machine learning approaches, and spatial representations of the data. We designed these use cases with the purpose of conducting analyses at the observation level (Use Case 1), patient cohorts (Use Case 2), and population-level data (Use Case 3).

**Results:**

For each use case, the results of the analyses were sufficiently statistically similar (*P* > 0.05) between the synthetic derivative and the real data to draw the same conclusions.

**Discussion and conclusion:**

This article presents the results of each use case and outlines key considerations for the use of synthetic data, examining their role in clinical research for faster insights and improved data sharing in support of precision healthcare.

**LAY SUMMARY**

Synthetic data enable data generation and sharing in support of research and precision healthcare. We demonstrated the creation and analysis of synthetic derivatives and validated the findings against original data. We used traditional statistics, machine learning approaches, and spatial representations of the data. For each use case, the results of the analyses were sufficiently similar—and statistically nonsignificant—between the synthetic derivative and the original data to draw the same conclusions. We outlined key considerations for the use of synthetic data, examining their role in clinical research for faster insights in support of precision healthcare.

## BACKGROUND AND SIGNIFICANCE

Large and comprehensive data sets are required to generate evidence in support of precision healthcare.^1^^,^^2^ However, current privacy and confidentiality controls surrounding clinical data which contain protected health information (PHI), such as the Common Rule^3^ and the HIPAA Privacy Rule,^4^ pose significant barriers to research using clinical data sets.^5^ We are at an inflection point regarding the availability of large data sets and require new approaches to addressing the protection of data privacy while rapidly advancing insights from health data.^6^

Ideally, synthetic data are nearly identical to original PHI data and can be analyzed as if they were original data but without any privacy concerns. Not only is there significant potential to protect patient privacy through analysis of data as a synthetic derivative,^7^ but synthetic derivatives of data can enable data sharing and accelerate discovery. Once real patient data are synthesized, the resulting data set no longer contains data on individual patients but rather is a collection of observations which maintain the statistical properties of the original data set. Since the data set no longer contains data on real patients, synthetic derivatives can be shared between researchers at different institutions. Further, time-to-insight can be shorter with the use of synthetic data derivatives due to reduced regulatory oversight. Since the data do not contain PHI, use of these data is not classified as human subjects research.

Data synthesis platforms are technologies that go beyond deidentification methods to produce data that can be used for research purposes and which obviate privacy and confidentiality concerns. Our institution partnered with MDClone (Beer Sheva, Israel) to evaluate their technology platform by developing and conducting three use cases. Each set of investigators conducted their research by analyzing real patient data and then repeating their analyses using synthetic data generated from the MDClone data synthesis platform. Collectively, we tested the robustness of the results using traditional statistics, machine learning approaches, and spatial representations of the data. Here, we present the results of these analyses and describe the strengths and limitations of using synthetic data for research.

## METHODS

Current state-of-the-art approaches for the generation of synthetic clinical data can be broadly classified as:

- Statistical simulation: where statistical models or profiles of normal human physiology and/or disease states are created based upon “real-world” data. Subsequently, these models or profiles are then used to create simulated patients or populations, consisting of “avatars” that “generate” clinical data consistent with the conditions and constraints of such models or profiles. As such, the ensuing simulated patients and their data are generally consistent with population-level norms and “look and feel” like “real-world” data;^8–10^ and
- Computational derivation: where a computational model of “real-world” data is produced on-demand, which can, in turn, be used to produce novel data in a multi-dimensional space (eg features) that adhere to the quantitative distributions and co-variance of the original source data. When using these types of models, data content and statistical features are a function of the input data set. This process can be repeated multiple times using the same source data and generating multiple derivative synthetic data sets. Further, such computationally derived synthetic data sets do not share mutual information with source data, eliminating reidentification potential.^1^^,^^11^

Of the above classifications, MDClone can be best described as using a computational derivation approach. Of note, the treatment of extreme values is handled by MDClone’s synthetic data engine to ensure that outlying individuals in the original data set will not be identifiable in the synthetic data derivative.

The MDClone data synthesis system includes three major components: a data lake, a query tool, and a synthetic data generator. The *data lake* can be used to integrate an arbitrary set of structured, semistructured, and unstructured data for subsequent analyses. Potential clinical data sources include, but are not limited to: inpatient and outpatient visits, medications, surgeries and procedures, laboratory tests, and demographic information. All data which are loaded in the data lake are stored with time stamps that are used to create a medical life story for each patient.

Events and attributes describing an event are identified by the researcher in the *query tool* according to the research question of interest. The reference, or index, event (ie a breast cancer diagnosis) defines the study population and is an anchor in the data set which provides the reference time stamp for all other events of interest (ie the first medication prescribed after the diagnosis of breast cancer). Properties of an event such as medication may include the medication name, dosage, or date dispensed, and determine the definition of the event (ie dosage greater than 750 mg/day). Commonly used hierarchies, such as those for discharge codes and medications, are employed to make identifying events more intuitive.

**Figure 1.**
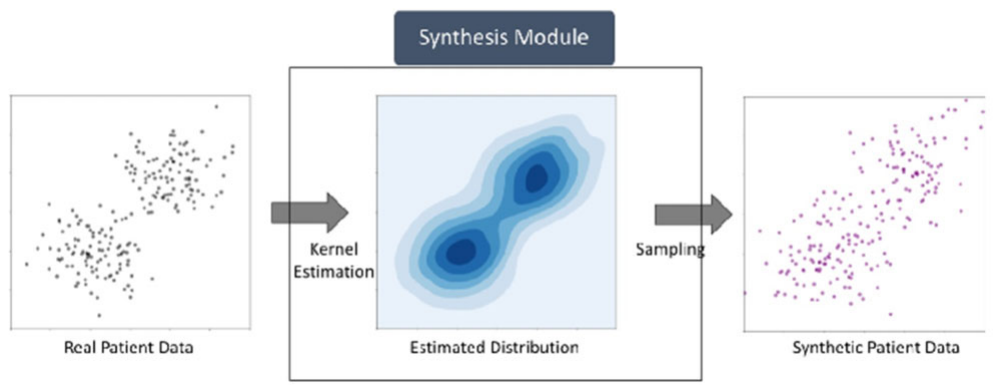
Data synthesis process.

The MDClone *synthetic data generator* transforms real patient data into a synthetic derivative which is statistically similar to the real patient data (Figure 1). The synthetic patient data are stored longitudinally based on the timestamp of each patient event. MDClone’s synthetic data generation pipeline consists of the following stages:

**Cohort identification**: In the first stage the user implements the inclusion and exclusion criteria for the study using various data filtration capabilities of the *query tool* such as “deceased males who were diagnosed with prostate cancer after January 1, 2010, before the age of 50.” Based on the criteria defined by the user, the cohort data are selected from the *data lake*.

**Feature selection**: After selecting the cohort, the next step is to select the features to be extracted for each patient in the cohort. This is achieved by using the *query tool* to select all the various attributes for each patient that will be needed to conduct the analysis. The user can also apply basic mathematical functions on the features including, but not limited to: max, median, variance, count, sum, etc. to form more advanced queries. The user can also define a more complex function, involving several properties (eg calculate a body mass index as the ratio of the first weight recorded after the diagnosis divided by the square of the first height recorded after the diagnosis). At the end of this stage, the user has a matrix of desired features from the *data lake* for patients selected in the cohort.

**Data synthesis**: The synthetic data generation is the most pivotal step in the pipeline. In this step, statistical models are created based on groups of similar patients using a variation of a kernel density estimation of the multivariate probability density (Figure 1). These models are then used to generate a synthetic cohort in a manner which preserves both the univariate and multivariate distributions of the underlying feature matrix created by the user at the end of the previous step. At the same time, irreversibility with respect to individual patients is maintained, since there is no correspondence between any members of the two cohorts.

**Summary analysis and report**: In the last stage, the MDClone system provides the user with an interface to visualize and validate the final output of the data synthesis module. It also generates a summary report at the end of the pipeline which shows a comparison between the real and synthetic data sets. The comparison report includes all pairwise correlations (real values versus synthetic data values for each feature) as well as a comparison of the statistical distributions of each feature in each data set.

MDClone's platform is a combination of an internal web application server, a batch process server, and a Hadoop cluster. The Hadoop cluster stores the organization's data in the data lake. This architecture enables data processing tasks to be broken down into smaller tasks that can be performed in parallel, thus allowing faster analytics on data which can contain billions of records. End users remain isolated from the original contents of the data lake and can query synthetic data derivatives from it via a virtualization server.

We designed use cases with the purpose of conducting analyses at the observation level (Use Case 1), with patient cohorts (Use Case 2), and population-level data (Use Case 3). We solicited these use cases from individuals already engaged in the use of our Research Data Core and who were working with the Clinical and Translational Science Award (CTSA)-funded Institute of Clinical and Translational Sciences (ICTS) in order to represent use cases across the translational spectrum.

All use case analyses were completed twice: first, using real patient data as extracted from the source systems, and second, using a platform-generated synthetic data set. These results allowed us to compare the results of analyses using MDClone’s data transformations to results from data extracted directly from the source systems. All statistical analyses on the original and synthetic data sets were done outside of the tool itself.

### Use Case 1: pediatric trauma

This use case was primarily a proof-of-concept for integrating retrospective clinical data with high-resolution data collected from patient bedside monitors. Clinical data included the Pediatric Risk of Mortality III (PRISM III),^12^ a measure used to predict the risk of mortality in critically ill children, and length of stay was obtained from a query of the St. Louis Children’s Hospital (SLCH), Virtual PICU Systems (VPS, LLC) Pediatric Intensive Care Unit (PICU) registry (www.myvps.org/vps-picu, last accessed November 14, 2020). High-resolution data including physiologic and alarm data from patient bedside monitors were extracted from the SLCH BedMaster high-resolution data repository (Excel Medical, Jupiter, FL, USA). Participants included in the analysis were pediatric trauma patients admitted to the PICU at SLCH from June 2014 through April 2018. Bedside monitor alarm data were captured using Alarm Navigator (Excel Medical, Jupiter, FL, USA), a system which archives data from the bedside monitoring system. Bedside alarm features included date, time, duration, severity, and standard description of alarms.

The focus of this use case was proof-of-concept testing whether high-resolution data could be loaded and generated from within the MDClone environment. Six features (PRISM score, PICU length of stay, count of alarms, count of alarms per length of stay, count of critical alarms, and count of critical alarms per length of stay) were used. The majority of data comprised continuous measurements (mortality prediction score, length of hospital stay, count of alarms per participant) and alarm severity was used to generate the “count of critical alarms” feature which had four levels (system, advisory, warning, crisis).

Four hundred and ten participants with 597 399 alarm events were included in the analysis. There were no missing data for this use case as all data elements were populated by the data collection systems while the patients were under investigation. Wilcoxon rank-sum tests were used to assess the differences in distributions between real and synthetic data. In addition, the relationship between PRISM III scores and number of alarms over PICU length of stay was assessed using Spearman rank correlation coefficients.

### Use Case 2: sepsis prediction

Sepsis is the dysregulated host response to infection that can result in life-threatening organ dysfunction. Over 1.7 million Americans develop sepsis every year and sepsis contributes to over a third of all in-hospital mortality cases.^13^^,^^14^ The ability to predict sepsis prior to onset would enable providers to take precautions or more closely monitor potentially septic patients so timely intervention could take place. To that end, we developed machine learning models to predict sepsis among general ward patients 6 h prior to onset. The goal of this use case was to evaluate if synthetic data could be used to train and develop predictive machine learning models which can be applied to real patients.

Electronic health record (EHR) data were collected from 2012 to 2017. Of 61 364 total inpatient encounters, we excluded those less than 12 h in duration, and those meeting the following criteria: sepsis was present on admission, no visit diagnosis codes were entered, insufficient vital sign data were obtained, or patients were admitted to the obstetrics and gynecology or psychiatric service. Features for predictive modeling included patient demographics, laboratory, and microbiological data. Sepsis cases (*n* = 643) were identified using the common interpretation of the Sepsis-2 criteria which comprised the systemic inflammatory response syndrome criteria and the suspicion of infection criteria.^15^ Nonsepsis cases (*n* = 1286) were sampled at a ratio of 1:2.

One hundred and sixty-nine features were included in the analysis that was either numerical or binary (categorical variables were transformed into one-hot binary vectors), such that the maximum cardinality for any categorical was technically 2, but would have been 4 for race (white, black, unknown, other).

Demographic characteristics of the cases and noncases were compared between real and synthetic data sets using Pearson’s chi-squared test (categorical), and Mann–Whitney’s rank-sum test (continuous). The sepsis logistic regression prediction model with L2-regularization was evaluated on training, 5-fold cross-validation, and 20% held-out test set for accuracy, precision, recall, F1-sore, and area under receiver operating curve (AUROC). A synthetic data set was generated based on the feature matrix. The model was trained and evaluated on the synthetic data set, the results of which were compared against a model trained and evaluated on real patient data. Also, we evaluated the model built on synthetic data with a real patient held-out test set to explore if models or conclusions drawn from synthetic data could be applicable to real patients.

### Use Case 3: public health dashboard

The purpose of the public health dashboard was to display clinical data by zip code in order to make the data actionable for stakeholders, such as public health and medical practitioners, researchers, and community members. It is often the case that community and civic organizations request clinical data in order to inform their public health promotion efforts in the community. In these situations, it is challenging to put in place the appropriate data sharing agreements in order to share real data and to maintain appropriate patient confidentiality. If synthetic data derivatives can be validly used for these types of data visualizations, doing so would also enable users to download the data from the dashboard to conduct their own analyses as the data would not contain PHI.

For these analyses, we calculated rates of chlamydia by year, using data from patients who tested positive for chlamydia in one of four emergency departments in our hospital system. We extracted data according to chlamydia diagnoses (binary) by year of the encounter (categorical) and zip code of patient residence (categorical). We additionally extracted the age of the patient (continuous) and patient sex and race (categorical).

Prior to data synthesis, we linked United States Census data by zip code to our EHR data to obtain measures of socioeconomic status as well as population counts to use as the denominator for the calculation of rates. Here we present unadjusted rates of infection per 100 000 persons and compare our findings between the real patient data and the synthetic data. In the future, our public health dashboard functionality will allow users to visualize rates of infections overall as well as to sort the data by year of diagnosis, patient demographics (ie age, gender, and race), and zip code level characteristics (ie percent below poverty). To demonstrate how well the synthetic data represented the real patient data graphically, we used Tableau 10.5 software to generate a map of the catchment area showing rates of chlamydia by zip code for a given year.

## RESULTS

### Use Case 1: pediatric trauma

The MDClone synthetic data process generated 401 participants, compared to 410 from the real data. It is important to note that synthetic data, unlike common methods which retain a one-to-one ratio between original and deidentified data sets, can produce any number of patients. MDClone's synthetic data engine produces a similar, but not necessarily identical, number of synthetic patients as in the original data set. Practically, this approach censors outliers for which there is insufficient signal in the original data to enable the generation of synthetic data. Of note, this is yet another layer of privacy protection, as sometimes the exact number of patients meeting an inclusion criteria, or the comparison between the number of patients meeting two slightly different inclusion criteria, may cause a privacy breach. We observed that the maximum values were generally lower among the variables in the synthetic data compared to the real data. However, the distributions of these variables did not differ between the real and synthetic data (all *P* = 0.9, Table 1).

**Table 1.** Five-figure distributions for PRISM III score, PICU length of stay and alarm data

	Real data						Synthetic data						
	(*N* = 410)						(*N* = 401)						
	Min	25th	50th	75th	90th	Max	Min	25th	50th	75th	90th	Max	*P* *
Prism III score	0	0	2	4	10.5	46	0	0	2	4	10	38	.93
Number of alarms	1	76	229	809	4840	17 688	6	80	236	792	4775	15 229	.99
ICU length of stay	0.1	0.7	1.3	3.4	10.6	73.3	0.2	0.7	1.3	3.1	9.5	34.3	.96
Number of alarms/ICU length of stay	2.9	70.6	168.5	422.6	737.0	2474.0	5.4	70.7	169.0	419.8	732.9	1496.2	.97

* Wilcoxon Rank Sum test comparing real versus synthetic data distributions.

**Figure 2.**
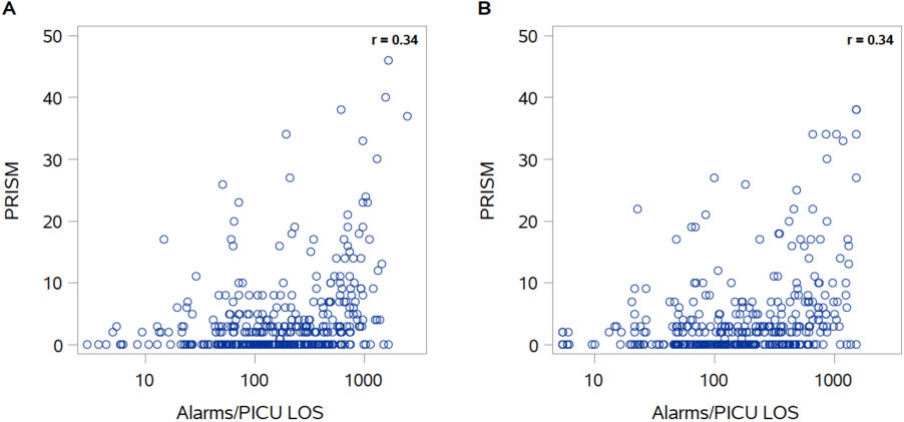
Alarms/PICU length of stay by PRISM III score: real (A) and synthetic (B) data.

In our real as well as our synthetic data, there was a low positive correlation between PRISM III scores and number of alarms experienced during the patient’s PICU stay (Spearman *r* = 0.34 for both). The comparison between real and synthetic data show similar patterns and correlation coefficients (Figure 2). Figure 2A represents a slightly different distribution of data as compared to Figure 2B. This difference is due to the data synthesis process, which depends upon a sufficient number of data points—which differs according to cohort size and requested features, in a particular position—in order to be carried out. If outliers are rare and thus cannot be synthesized in the resulting data set, these observations are censored in order to maintain data privacy.

### Use Case 2: sepsis prediction

For the sepsis cases, time of sepsis onset served as the index time. For the nonsepsis cases, if the patient was admitted for greater than 24 h, the index time was the midpoint between admission and discharge. Otherwise, the index time was designated as 12 h into admission. Features were constructed from data 6 to 24 h prior to the index time. Features (*n* = 169) used for prediction included: patient demographics, lab results, vital signs, and admission characteristics. Longitudinal data were converted into sets of unitary statistical values (eg mean, standard deviation, last, etc.). Lab results were converted into sets of binary abnormal flags (eg low, high, or critical lactate). The resulting feature matrix was used in the MDClone platform to generate a synthetic data set. Demographic data comparing real and synthetic data sets are displayed in Table 2. We made these comparisons using a chi-squared test for categorical variables and Kolmogorov–Smirnov test for continuous variables. The distribution of demographic variables was similar between real and synthetic sepsis populations and between real and synthetic nonsepsis populations.

**Table 2.** Demographic characteristics and comorbidities (mean, SD, or *N*, %, and *P*-values) of the sepsis and nonsepsis cases: real versus synthetic data sets

			Real nonsepsis	Synth sepsis	Synth nonsepsis	Real sepsis vs. synth sepsis	Real nonsepsis vs. Synth nonsepsis	Real sepsis vs. real nonsepsis	Synth sepsis vs. synth nonsepsis
Variable category	Variable	Real sepsis	*n* (%) for categorial data Mean ± SD for continuous data				*P*-value Chi-squared test for categorical data Kolmogorov–Smirnov (K-S) 2-sample test for continuous data			
Demographics	Age	60.28 ± 15.19	59.06 ± 17.54	60.30 ± 15.12	58.98 ± 17.52	1.000	1.000	.080	.050
	Male	278 (54.4%)	281 (54.6%)	465 (26.9%)	466 (27.1%)	.991	.977	.001	.000
	Race: Caucasian	374 (73.2%)	378 (73.4%)	692 (36.2%)	692 (36.5%)	.996	.962	.017	.010
	Race: Black	95 (18.6%)	95 (18.4%)	304 (9.2%)	306 (9.2%)	.983	.996	.000	.000
	Race: Unknown	18 (3.5%)	18 (3.5%)	17 (1.7%)	17 (1.7%)	.884	.869	.032	.033
	Race: Other	24 (4.7%)	24 (4.7%)	19 (2.3%)	19 (2.3%)	.904	.877	.002	.002
	Admitted through ER	119 (23.3%)	119 (23.1%)	433 (11.5%)	434 (11.5%)	.996	.974	.000	.000
	ICU stay prior to prediction period	25 (4.9%)	25 (4.9%)	15 (2.4%)	15 (2.4%)	.907	.860	.000	.000
	30-day readmission cases	20 (3.9%)	20 (3.9%)	43 (1.9%)	43 (1.9%)	.892	.923	.921	.906
Vital signs (mean value during prediction period)	Temperature	36.29 ± 0.44	36.20 ± 0.32	36.29 ± 0.43	36.20 ± 0.30	.840	.160	.000	.010
	Respiratory rate	18.51 ± 2.13	17.79 ± 1.42	18.48 ± 1.99	17.81 ± 1.42	.430	.310	.000	.000
	Heart rate	85.29 ± 17.14	78.51 ± 13.27	85.17 ± 16.98	78.50 ± 13.19	.900	1.000	.000	.000
	SpO2	96.47 ± 2.42	97.12 ± 1.89	96.52 ± 1.97	97.12 ± 1.88	.990	.670	.000	.000
	Systolic blood pressure	126.3 ± 18.3	128.5 ± 18.1	126.3 ± 18.3	128.5 ± 18.1	.940	.990	.010	.040
	Diastolic blood pressure	70.22 ± 12.01	70.75 ± 11.52	70.11 ± 12.06	70.75 ± 11.46	.990	.990	.610	.400
	Shock index	0.692 ± 0.169	0.625 ± 0.142	0.691 ± 0.165	0.624 ± 0.142	.980	.920	.000	.000
Abnormal vital sign readings	Heart rate	278 (54.4%)	279 (54.2%)	372 (26.9%)	372 (27.0%)	.991	.997	.000	.000
	Respiratory rate	90 (17.6%)	90 (17.5%)	91 (8.7%)	91 (8.7%)	.980	.954	.000	.000
	Systolic blood pressure	37 (7.2%)	38 (7.4%)	34 (3.6%)	34 (3.7%)	.972	.912	.001	.001
	Diastolic blood pressure	225 (44.0%)	229 (44.5%)	454 (21.8%)	455 (22.1%)	.938	.976	.968	.893
	SpO2	157 (30.7%)	159 (30.9%)	209 (15.2%)	209 (15.4%)	.987	.983	.000	.000
	Temperature	21 (4.1%)	21 (4.1%)	15 (2.0%)	15 (2.0%)	.895	.860	.002	.002
Abnormal lab results	Albumin	53 (10.4%)	53 (10.3%)	65 (5.1%)	65 (5.1%)	.952	.941	.006	.007
	BUN	100 (19.6%)	101 (19.6%)	171 (9.7%)	172 (9.8%)	.951	.977	.166	.166
	Bilirubin	36 (7.0%)	36 (7.0%)	43 (3.5%)	43 (3.5%)	.930	.923	.022	.023
	Calcium	83 (16.2%)	83 (16.1%)	137 (8.0%)	137 (8.0%)	.976	.969	.136	.146
	Chloride	49 (9.6%)	51 (9.9%)	58 (4.7%)	58 (4.9%)	.949	.937	.005	.003
	Creatinine	135 (26.4%)	136 (26.4%)	213 (13.1%)	213 (13.1%)	.947	.983	.013	.012
	Glucose	28 (5.5%)	28 (5.4%)	33 (2.7%)	33 (2.7%)	.914	.910	.043	.045
	Hematocrit	224 (43.8%)	225 (43.7%)	343 (21.7%)	343 (21.7%)	.987	1.000	.000	.000
	Hemoglobin	200 (39.1%)	201 (39.0%)	363 (19.4%)	363 (19.4%)	.978	.998	.143	.142
	INR	69 (13.5%)	71 (13.8%)	65 (6.7%)	65 (6.9%)	.967	.941	.000	.000
	Potassium	35 (6.8%)	36 (7.0%)	62 (3.4%)	62 (3.5%)	.973	.939	.596	.515
	Lactate	7 (1.4%)	7 (1.4%)	3 (0.7%)	3 (0.7%)	.799	.685	.032	.032
	Sodium	61 (11.9%)	62 (12.0%)	68 (5.9%)	68 (6.0%)	.963	.943	.001	.000
	PaCO2	25 (4.9%)	25 (4.9%)	6 (2.4%)	6 (2.4%)	.907	.776	.000	.000
	PaO2	69 (13.5%)	70 (13.6%)	8 (6.7%)	8 (6.8%)	.961	.806	.000	.000
	Platelet	65 (12.7%)	66 (12.8%)	94 (6.3%)	94 (6.4%)	.962	.956	.035	.029
	RBC	192 (37.6%)	194 (37.7%)	332 (18.6%)	332 (18.7%)	.974	.999	.040	.033
	Troponin	7 (1.4%)	7 (1.4%)	23 (0.7%)	23 (0.7%)	.799	.889	.340	.334
	WBC	121 (23.7%)	123 (23.9%)	176 (11.7%)	177 (11.9%)	.997	.976	.002	.002
Elixhauser comorbidities	Chronic pulmonary disease	189 (37.0%)	191 (37.1%)	190 (18.3%)	191 (18.5%)	.975	.975	.000	.000
	Coagulopathy	139 (27.2%)	139 (27.0%)	44 (13.5%)	44 (13.4%)	.995	.924	.000	.000
	Congestive heart failure	178 (34.8%)	179 (34.8%)	125 (17.2%)	125 (17.3%)	.968	.966	.000	.000
	Diabetes, complicated	70 (13.7%)	70 (13.6%)	68 (6.8%)	68 (6.8%)	.967	.943	.000	.000
	Diabetes, uncomplicated	91 (17.8%)	92 (17.9%)	196 (8.8%)	196 (8.9%)	.954	.981	.622	.658
	Fluid and electrolyte disorders	359 (70.3%)	362 (70.3%)	238 (34.8%)	238 (35.0%)	.956	.987	.000	.000
	Hypertension, complicated	103 (20.2%)	104 (20.2%)	103 (10.0%)	103 (10.0%)	.950	.959	.000	.000
	Hypertension, uncomplicated	220 (43.1%)	221 (42.9%)	414 (21.3%)	417 (21.4%)	.986	.972	.294	.351
	Hypothyroidism	75 (14.7%)	75 (14.6%)	145 (7.3%)	145 (7.2%)	.971	.971	.799	.828
	Liver disease	63 (12.3%)	65 (12.6%)	62 (6.1%)	62 (6.3%)	.962	.939	.000	.000
	Lymphoma	10 (2.0%)	10 (1.9%)	11 (1.0%)	11 (1.0%)	.835	.836	.235	.239
	Metastatic cancer	32 (6.3%)	32 (6.2%)	70 (3.1%)	70 (3.1%)	.923	.944	.781	.762
	Obesity	121 (23.7%)	121 (23.5%)	132 (11.7%)	132 (11.7%)	.997	.968	.000	.000
	Peripheral vascular disorders	98 (19.2%)	99 (19.2%)	92 (9.5%)	92 (9.6%)	.951	.955	.000	.000
	Pulmonary circulation disorders	91 (17.8%)	92 (17.9%)	42 (8.8%)	42 (8.9%)	.954	.922	.000	.000
	Renal failure	131 (25.6%)	132 (25.6%)	184 (12.7%)	185 (12.8%)	.944	.975	.000	.000
	Solid tumor without metastasis	54 (10.6%)	54 (10.5%)	155 (5.2%)	155 (5.2%)	.953	.973	.020	.018
	Valvular disease	169 (33.1%)	169 (32.8%)	81 (16.4%)	81 (16.3%)	.983	.950	.000	.000
	Elixhauser comorbidity score	15.01 ± 10.76	6.52 ± 8.12	14.90 ± 10.72	6.49 ± 8.14	.620	.950	.000	.000

Next, we evaluated if models trained on synthetic data and tested on real data would perform equivalently to models trained on real data and tested on real data (as well as models trained on synthetic data and tested on synthetic data). Performance metrics for all models were similar, as shown in Table 3.

**Table 3.** Performance metrics of sepsis prediction models

Training set		Real	Synthetic	Synthetic
Testing set		Real	Synthetic	Real
**Train**	**Accuracy**	0.845	0.869	0.852
	**Precision**	0.803	0.840	0.812
	**Recall**	0.704	0.758	0.719
	**F1**	0.750	0.797	0.763
	**AUROC**	0.809	0.842	0.818
**5-fold cross-validation**	**Accuracy**	0.795	0.802	0.799
	**Precision**	0.712	0.73	0.723
	**Recall**	0.637	0.67	0.639
	**F1**	0.672	0.69	0.678
	**AUROC**	0.855	0.86	0.847
**Test**	**Accuracy**	0.834	0.833	0.834
	**Precision**	0.811	0.759	0.829
	**Recall**	0.677	0.678	0.654
	**F1**	0.738	0.716	0.731
	**AUROC**	0.887	0.885	0.892

### Use Case 3: public health dashboard

Cases of chlamydia corresponded to patients who were predominantly female with an average age of 23 years (Table 4). Across all years of observation, rates of chlamydia ranged from 80 to 105 cases per 100 000 population. We see from Table 4 that the average age, gender distribution, and chlamydia rates are consistent between the real patient data and the synthetic data. The number of chlamydia cases per 100 000 population per year did not differ significantly between the real and synthetic data sets (data not shown).

**Table 4.** Characteristics (mean, SD, or *N*, %) of patients and rates of chlamydia (per 100 000 persons) by year: real versus synthetic data

	Real				Synthetic			
Year	*N*	Rate	Age (years)	Female	*N*	Rate	Age (years)	Female
2010	456	78.5	22.5 (5.2)	316 (69.3)	475	78.5	22.5 (5.1)	328 (69.1)
2011	475	83.6	22.8 (6.2)	321 (67.6)	475	80.2	22.6 (5.5)	321 (67.6)
2012	541	105.5	23.2 (5.7)	368 (68.0)	525	101.2	23.1 (5.6)	358 (68.2)
2013	469	92.2	23.2 (6.2)	292 (62.3)	475	91.5	23.3 (6.3)	297 (62.5)
2014	459	96.3	23.4 (5.5)	266 (58.0)	450	93.3	23.4 (5.5)	260 (57.8)
2015	505	86.6	23.4 (5.7)	289 (57.2)	525	88.7	23.3 (5.7)	297 (56.6)

**Figure 3.**
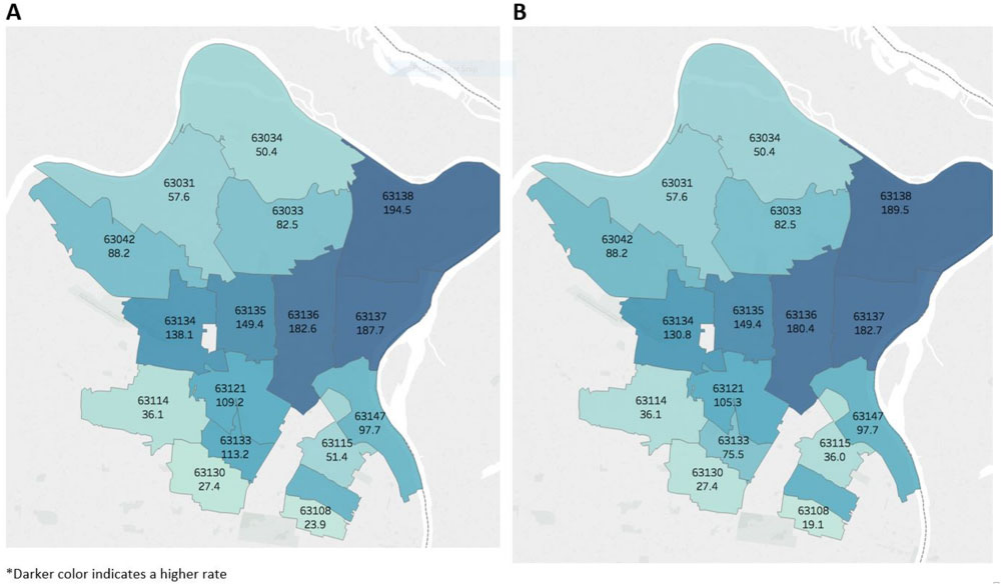
Chlamydia rates (per 100 000 persons) by zip code: real (left) versus synthetic (right) data, 2014. *Darker color indicates a higher rate.

A graphical comparison of chlamydia rates in the year 2014, between the real and synthetic data, is shown in Figure 3. Zip codes with higher rates of infection are shaded darker blue, while lower rates of infection are indicated by lighter shades of blue. Each geographic unit is labeled with its respective zip code and chlamydia rate for that year, and the results are not clinically or statistically distinguishable from each another (all *P* > 0.05). In fact, the rate differences and 95% confidence intervals for each year, comparing real to synthetic data sets, are as follows: 2010 (0); 2011 (−3.4, −14.5 to 7.8); 2012 (−4.3, −17.4 to 8.8); 2013 (−0.7, −13.3 to 11.9); 2014 (−2.9, −16.2 to 10.4); and 2015 (2.1, −9.3 to 13.5).

## DISCUSSION

Overall, we demonstrated that high-resolution alarm, clinical, and geographic data can be combined and successfully extracted and analyzed using the MDClone platform. We used distinct data sets and methodologies for each use case in order to evaluate the reliability of the data synthesis platform to yield data sets with the same or similar statistical output as the real data sets. In each use case, the results of the analyses were sufficiently similar (*P* > 0.05) between the synthetic derivative and the real data to draw the same conclusions according to the subject matter experts consulted for each use case. In several instances, the results were exactly the same, and rarely were there statistically significant differences between data sets. Parity among performance metrics across the different train-test combinations in the sepsis use case suggests that synthetic data can be used to construct prediction models for use on real patients.

There were no missing data in Use Case 1 or meaningful missingness in Use Case 3 which allowed for a comprehensive data set to be analyzed in each of these scenarios. The only challenge faced in this regard for the sepsis use case (Use Case 2) was converting longitudinal data into a format that could be used to generate synthetic data. Low sample size, high sparsity, high dimensionality, and highly irregular distributions can all affect the data synthesis process as well as the interpretability of real data.

The challenges of building meaningful cohorts and protecting privacy are interconnected and cannot be separated for the type of next-generation research we conducted.^7–10^^,^^16^^,^^17^ Many current data-anonymization techniques rely on data manipulation concepts such as aggregation (associating a higher category to some of the features in order to generalize them), subsampling from a larger population in order to achieve the final desired population size, and adding noise to the data set. However, the usefulness of the data set resulting from the above techniques is questionable, and they are not usually safeguarded against reidentification. Other approaches build generative models from the original data in order to imitate its statistical characteristics, allowing for advanced data analysis without compromising individuals' privacy. However, these algorithms often require assumptions about the specific shapes of the original distributions, which could in fact be very complex or nonparametric.

MDClone's synthetic data engine is based on a different approach that does not focus on camouflaging individual patients. With MDClone, models are created based on groups of similar patients. These models are used to create new “patients,” and the resulting distinction between the synthetic population and the original true population assures irreversibility. The process is on-demand, and cohorts can be altered (ie new features added) and data can be changed (ie adding or replacing variables) easily, which provides the benefit of faster research cycles. In this way, synthetic data generation enables repeated queries of the data, as synthetic patients are not original patients masked by noise.

In contrast, the differential privacy approach adds noise to the data to ensure privacy, and an adequate threshold for such noise remains unknown. There may be an ideal amount of noise added for a single query, yet querying the data multiple times may result in insufficient privacy protections. Further, MDClone is not susceptible to a model inversion attack as described by Veale et al.;^16^ the synthetic data derivative does not contain a one-to-one ratio between the training set B and the members of B′, which is created from a model that is not based on a specific member of B.

A recently published paper outlines an additional five use cases for the application of MDClone-generated synthetic data derivatives to research questions, including an exploration of how the generation of several MDClone data derivatives from the same query performs satisfactorily over multiple iterations.^16^ Our study adds to the growing literature of synthetic data validation in the following ways: (1) providing a demonstration of generating synthetic data derivatives from high-fidelity data, (2) deployment of machine learning approaches designed to test the fit-for-purpose of using synthetic data as the training data for prediction models, and (3) determining the utility of synthetic data for geospatial analyses.

Other commercial systems and approaches to synthetic data generation^9^^,^^10^^,^^17^^,^^18^ may depend on demographics most accurately representing the use case and the current knowledge available. Thus, novel insights may be limited by the typical outcomes observed given the demographics of the cohort as well as by prespecified relationships between variables which dictate their inclusion or exclusion in the resulting data set.^9^ Therefore, other commercial systems may not be flexible with respect to diverse use cases nor allow for clinical discovery.^10^

Alternative methods of synthetic data generation tend to fall short of preserving complex nonlinear relations and handling the “messy” nature of real-world clinical data. Of note, MDClone does not use the common kernel density estimation approach in isolation, as kernel density estimation alone would not provide the required outcome from a privacy standpoint. The utility of synthetic data for predictive modeling in healthcare should continue to be explored and a comparison of approaches will help the research community define which approaches are fit-for-purpose with respect to particular use cases. For relatively rare conditions, one site may not have sufficient data to train a predictive model, yet a consortium of institutions contributing synthetic data might have sufficient numbers of patients to make meaningful inferences.

For these analyses, we used a *P*-value threshold of .05 to maintain simplicity of presenting results from multiple use cases. We acknowledge that such thresholds would (and should) vary by use case and specifically by the amount of error a researcher is willing to tolerate given the context of the research question.

A key enabling feature for the construction of reliable synthetic data is choosing a large yet finite list of columns. It would not be possible to synthesize the entire EHR as a one-time process, as there are many more features than patients. Thus, choosing a list of features to be used in the analysis—where the number of features may be large but still much smaller than the number of patients in the cohort—enables the creation of reliable synthetic data.

## CONCLUSIONS

We conclude that the potential for leveraging synthetic data for the conduct of research is great; its use empowers researchers to produce valid results, over a short period of time, while protecting patient privacy. It is expected that the analysis of synthetic data will accelerate the conduct of data-driven research studies as protocols will not be required to be IRB-reviewed since the research is not classified as that of human subjects and the data do not contain PHI. Further, we anticipate that generating synthetic data derivatives for research will reduce barriers to data sharing, which have traditionally included concerns about data privacy and ownership.

In addition to the use cases presented herein, other applications of synthetic data include the ability to conduct studies of varied designs (ie case–control) as well as assessments of patient safety and care quality. Future directions for this work include developing and validating clinical risk prediction models, sharing data across institutions and countries, enabling open competitions for data mining, and evaluating drug responsiveness.^1^

## CLINICAL RELEVANCE STATEMENT

Data synthesis platforms like MDClone are expected to dramatically enhance the research community’s ability to use clinical data for faster insights and improved data sharing in support of precision healthcare.
